# Activation of the *Listeria monocytogenes* Virulence Program by a Reducing Environment

**DOI:** 10.1128/mBio.01595-17

**Published:** 2017-10-17

**Authors:** Jonathan L. Portman, Samuel B. Dubensky, Bret N. Peterson, Aaron T. Whiteley, Daniel A. Portnoy

**Affiliations:** a Graduate Group in Infectious Diseases and Immunity, School of Public Health, University of California, Berkeley, Berkeley, California, USA; b Department of Molecular and Cell Biology, University of California, Berkeley, Berkeley, California, USA; c Graduate Group in Microbiology, University of California, Berkeley, Berkeley, California, USA; d School of Public Health, University of California, Berkeley, Berkeley, California, USA; UCLA School of Medicine

**Keywords:** glutathione, c-di-AMP, gram-positive bacteria, stringent response, virulence, virulence regulation

## Abstract

Upon entry into the host cell cytosol, the facultative intracellular pathogen *Listeria monocytogenes* coordinates the expression of numerous essential virulence factors by allosteric binding of glutathione (GSH) to the Crp-Fnr family transcriptional regulator PrfA. Here, we report that robust virulence gene expression can be recapitulated by growing bacteria in a synthetic medium containing GSH or other chemical reducing agents. Bacteria grown under these conditions were 45-fold more virulent in an acute murine infection model and conferred greater immunity to a subsequent lethal challenge than bacteria grown in conventional media. During cultivation *in vitro*, PrfA activation was completely dependent on the intracellular levels of GSH, as a glutathione synthase mutant (Δ*gshF*) was activated by exogenous GSH but not reducing agents. PrfA activation was repressed in a synthetic medium supplemented with oligopeptides, but the repression was relieved by stimulation of the stringent response. These data suggest that cytosolic *L. monocytogenes* interprets a combination of metabolic and redox cues as a signal to initiate robust virulence gene expression *in vivo*.

## IMPORTANCE

Intracellular pathogens are responsible for much of the worldwide morbidity and mortality from infectious diseases. These pathogens have evolved various strategies to proliferate within individual cells of the host and avoid the host immune response. Through cellular invasion or the use of specialized secretion machinery, all intracellular pathogens must access the host cell cytosol to establish their replicative niches. Determining how these pathogens sense and respond to the intracellular compartment to establish a successful infection is critical to our basic understanding of the pathogenesis of each organism and for the rational design of therapeutic interventions. *Listeria monocytogenes* is a model intracellular pathogen with robust *in vitro* and *in vivo* infection models. Studies of the host-sensing and downstream signaling mechanisms evolved by *L. monocytogenes* often describe themes of pathogenesis that are broadly applicable to less tractable pathogens. Here, we describe how bacteria use external redox states as a cue to activate virulence.

## INTRODUCTION

The facultative intracellular pathogen *Listeria monocytogenes* is the third leading cause of death from foodborne illness in the United States, with an estimated 1,600 cases leading to 260 deaths per year (1). This ubiquitous Gram-positive saprophyte is found in soil, where it commonly contaminates produce and livestock products like dairy milk (2). Upon ingestion by, primarily, immunocompromised individuals and pregnant women, the bacteria traverse the intestinal epithelium and cause systemic infection, often leading to miscarriage, neonatal sepsis, or meningitis (3). After invasion, a critical regulatory switch occurs during the transition from vacuole to cytosol, when *L. monocytogenes* significantly remodels its transcriptional profile by activating the master virulence regulator PrfA (4, 5). Proper temporal expression of PrfA is critical for bacterial invasion and vacuolar escape, as inappropriate expression leads to a loss of fitness both in and out of the host (6, 7).

PrfA is directly responsible for the transcription of 10 core virulence genes and indirectly affects the expression of over 140 others, many of which are essential for virulence (8). The activity of PrfA is tightly regulated and only becomes activated upon entry into cells. This strict regulation is responsible for the transcript levels of the PrfA-dependent actin assembly-inducing protein ActA increasing over 200-fold in the host cytosol compared to its levels in broth cultures (9, 10). While the precise cues that define the intracellular milieu are not described and complete activation of PrfA-mediated gene expression has not been recapitulated *in vitro*, it is clear that PrfA activity is allosterically activated by the small molecule glutathione (GSH) (11, 12). GSH is a tripeptide antioxidant canonically utilized by eukaryotes, cyanobacteria, and proteobacteria as a redox buffer to protect against oxidative damage (13). *L. monocytogenes* is unusual in that it also synthesizes GSH (14). During infection, the expression of the bacterial glutathione synthase gene (*gshF*) increases 10-fold; however, it is still not appreciated why *gshF* is upregulated in host cells or why exogenous GSH is insufficient to activate PrfA in traditional broth culture (11; J. L. Portman and D. A. Portnoy, unpublished data).

A number of diverse factors influence PrfA activity, including temperature, osmolarity, and iron availability (5, 15). Furthermore, perturbations of several metabolic pathways influence the activity of PrfA *in vitro* (16), including those that influence the pleiotropic metabolic repressor CodY. CodY regulates hundreds of genes in response to nutrient starvation and directly interacts with the coding region of the *prfA* gene (17–19). In addition to sensing intracellular levels of branched-chain amino acids (BCAAs), the activity of CodY is also influenced by intracellular GTP pools, which can be quickly depleted during starvation upon the production of the nucleotide secondary messengers guanosine penta- and tetraphosphate [combined here and referred to collectively as (p)ppGpp] (20–22) in a process known as the stringent response. Due to the direct relationship between CodY and *prfA* (17), we hypothesized that manipulating CodY via (p)ppGpp through growth on a specialized medium may be required for PrfA activation by GSH *in vitro*.

Here, we report that the growth of *L. monocytogenes* in nutrient-limiting synthetic medium is sufficient to allow robust activation of PrfA by exogenous glutathione and chemical reducing agents. Our findings help to unify the results of a number of PrfA-related studies into a two-step activation model and clearly demonstrate the potent regulatory role of metabolic signaling in virulence gene regulation in *L. monocytogenes*. These findings may help explain how *L. monocytogenes* senses entry into the host cytosolic compartment to appropriately upregulate PrfA-dependent virulence gene expression.

## RESULTS

### Glutathione is sufficient to activate PrfA in synthetic but not rich medium.

**FIG 1:**
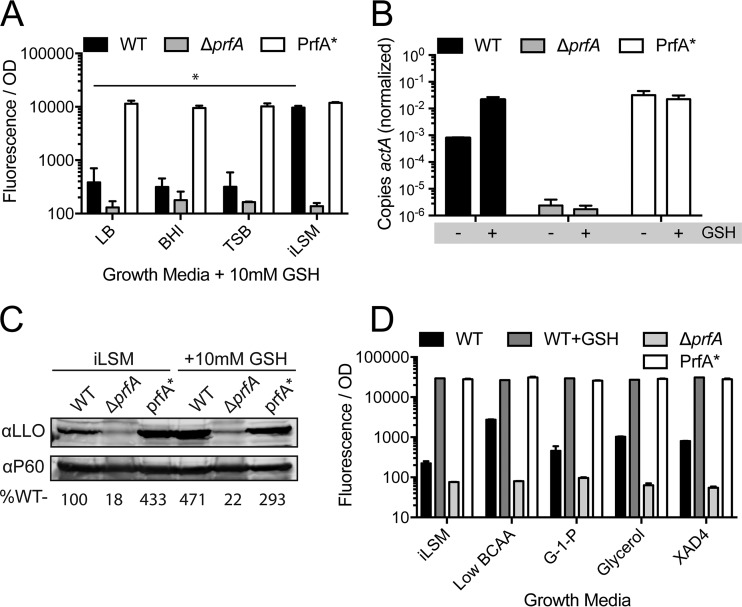
Exogenous glutathione is sufficient to activate PrfA *in vitro*. (A) Strains of *L. monocytogenes* expressing RFP under the control of the PrfA-dependent *actA* promoter (P_*actA*__RFP) were grown in various media containing 10 mM glutathione (GSH). *, *P* < 0.05. (B) Strains of *L. monocytogenes* were grown to mid-log phase in iLSM medium in the presence or absence of 10 mM GSH. Total RNA was harvested, and transcript abundance of *actA* was quantified using RT-qPCR and normalized to that of the housekeeping gene *bglA*. *, *P* < 0.05. (C) Strains of *L. monocytogenes* were grown to stationary phase in iLSM medium in the presence or absence of 10 mM GSH. Precipitated culture supernatants were separated by SDS-PAGE and probed for listeriolysin O (LLO) by Western blotting. The autolysin P60 was also quantified and used as a loading control. The values reported are the abundances of LLO/P60 relative to the levels in the wild type, as percentages. (D) Strains of *L. monocytogenes* expressing P_*actA*__RFP were grown in iLSM or variations thereof with 20% standard BCAA (Low BCAA), glucose-1-phosphate substituted for glucose (G-1-P), glycerol substituted for glucose (Glycerol), or iLSM with 1% (wt/vol) Amberlite XAD-4 resin (XAD4). Fluorescence was measured and normalized to the bacterial number using the respective OD_600_. Error bars show standard deviations. WT, wild type; *, *P* < 0.05.

In order to rapidly monitor PrfA activity under various conditions, a strain of *L. monocytogenes* was utilized that expresses red fluorescent protein (RFP) under the control of the tightly regulated PrfA-dependent promoter for the actin polymerization gene, *actA* (10, 23, 24). This transcriptional fusion was used to test the ability of exogenous GSH to activate PrfA during *in vitro* growth under various conditions and in various culture media. The growth of *L. monocytogenes* in all standard growth media showed negligible increases in fluorescence upon the addition of 10 mM GSH. However, growth in a defined synthetic medium (iLSM) (56), led to a higher basal level of fluorescence, as well as a robust increase of fluorescence in response to GSH (Fig. 1A) (a dose-dependent response to GSH is also shown by the results in Fig. 4C). The level of PrfA activation seen in iLSM supplemented with GSH (iLSM-GSH) was equal to that seen with a variant of PrfA, hereinafter called PrfA*, that is locked in the active conformation [PrfA*, encoded by the allele *prfA*(G145S), bears a G-to-S change at position 145] (25). To verify that the transcriptional fusion was accurately reporting PrfA-dependent activity, direct mRNA quantification of the *actA* gene was performed using quantitative reverse transcription PCR (RT-qPCR) (Fig. 1B) and the protein secretion of another PrfA-regulated virulence factor, listeriolysin O (LLO), was measured by Western blotting (Fig. 1C) (26, 27). In all scenarios, the addition of GSH to iLSM was sufficient to induce PrfA activation to levels comparable to those seen for PrfA*.

To compare the effect of GSH to those of other inducers of PrfA reported in the literature, we adapted our synthetic medium to test growth with glycerol, low concentrations of BCAAs, l-glutamine, the phosphosugar glucose-1-phosphate and in the presence of the charcoal-like resin XAD-4 (17, 28–33). Consistent with previous reports, each of these growth conditions led to an increase in fluorescence from the reporter strain, but only iLSM-GSH induced activation equal to that of the PrfA* control (Fig. 1D). In addition, various concentrations of glucose and the disaccharide cellobiose were tested for their reported ability to repress PrfA activity (34, 35); however, under all conditions tested, GSH fully activated PrfA (J. L. Portman and D. A. Portnoy, data not shown).

### Nutritive oligopeptides potently inhibit PrfA activation by glutathione.

**FIG 2:**
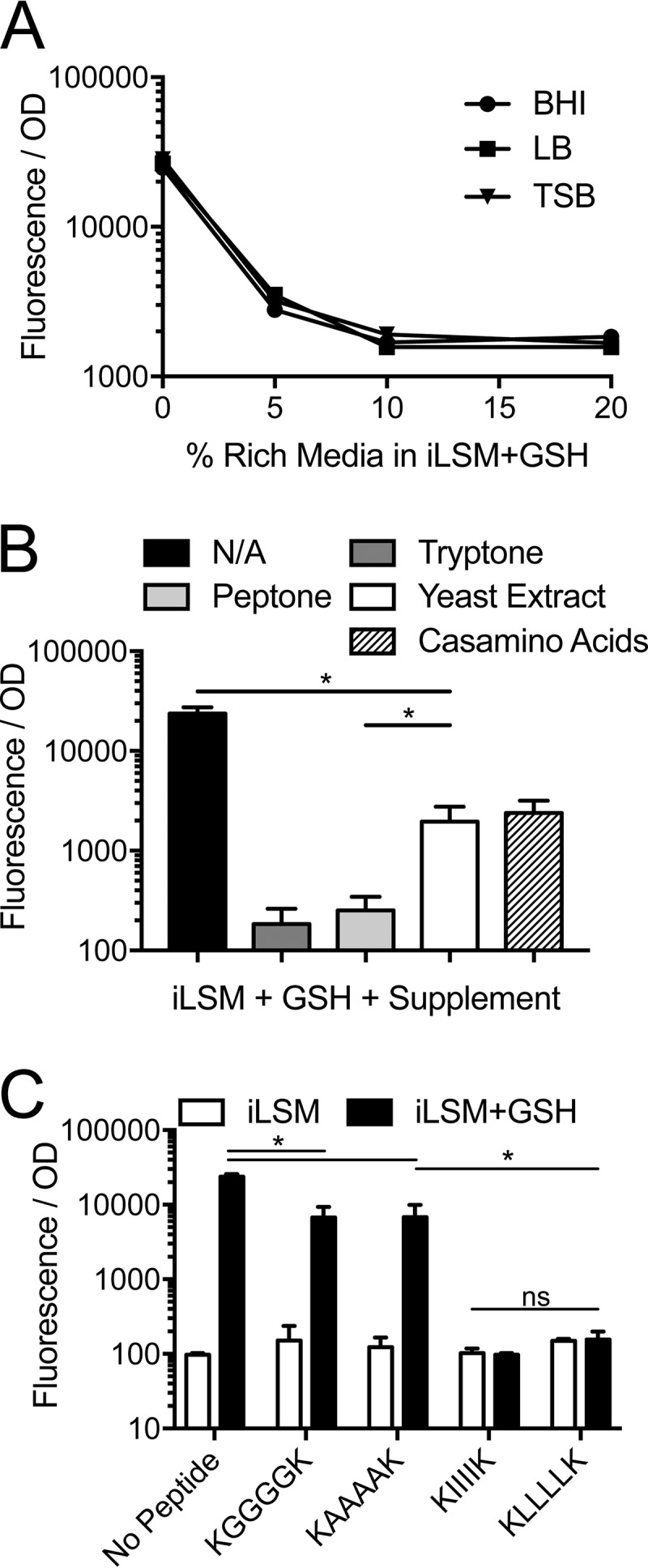
Oligopeptides inhibit activation of PrfA *in vitro*. Wild-type *L. monocytogenes* expressing P_*actA*__RFP was grown in a mixture of iLSM and rich medium plus 10 mM GSH (A), iLSM with various supplements plus 10 mM GSH (B), or iLSM with or without synthetic peptides and with or without 10 mM GSH (C). Fluorescence was measured and normalized to the bacterial number using the respective OD_600_. N/A denotes iLSM with no additions. Error bars show standard deviations. *, *P* < 0.05.

In order to differentiate between the presence of PrfA-activating components specific to iLSM and the presence of inhibitory components in rich media, iLSM was mixed with rich media at different ratios and tested for the ability of exogenous GSH to activate PrfA. Consistent with the notion that rich media contain inhibitory molecules, approximately 8% of any of the three rich media tested was sufficient to completely block the activation of PrfA by GSH (Fig. 2A). To determine what components of rich media were responsible for the inhibition, common ingredients of rich media, including tryptone, peptone, yeast extract, and Casamino acids, were tested. When added to iLSM-GSH at the concentrations found in standard LB medium, yeast extract and Casamino acids had negligible effects on PrfA activation, while tryptone and peptone potently repressed its activation (Fig. 2B). These data suggested that oligopeptides found in tryptone and peptone (and to a much lesser degree, yeast extract and Casamino acids), were primarily responsible for the potent inhibition of PrfA activation by GSH *in vitro*. To confirm that these oligopeptides were sufficient for repression, synthetically derived hexapeptides containing repeats of glycine, alanine, leucine, or isoleucine with flanking lysines (for solubility) were tested in iLSM-GSH (Fig. 2C). While the glycine- and alanine-containing peptides yielded significant but incomplete repression, peptides containing leucine and isoleucine potently inhibited PrfA activation. These data suggest that oligopeptides containing BCAAs are potently inhibitory to PrfA activation by GSH *in vitro* and are consistent with findings that limiting the amounts of BCAAs in minimal medium leads to increased basal levels of PrfA activity (Fig. 1D) (17).

### Induction of the stringent response is sufficient to rescue PrfA activation by GSH in the presence of oligopeptides.

**FIG 3:**
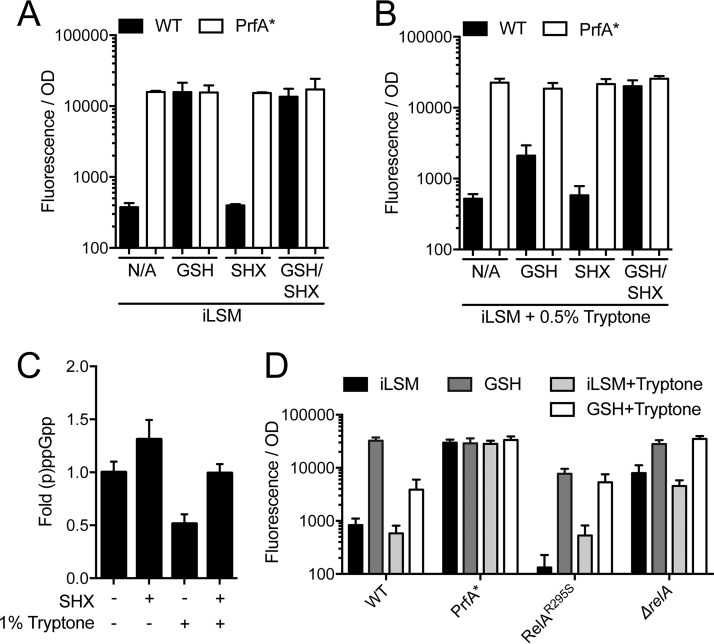
Increasing intracellular (p)ppGpp reverses the inhibitory effect of oligopeptides. (A and B) Wild-type or PrfA* *L. monocytogenes* expressing P_*actA*__RFP was grown in iLSM (A) or iLSM plus 0.5% tryptone (B) and supplemented with 10 mM GSH, 2 mg/ml dl-serine hydroxamate (SHX), or both. Fluorescence was measured and normalized to the bacterial number using the respective OD_600_. *, *P* < 0.05. (C) Quantification of ^32^P-labeled (p)ppGpp from bacteria. Wild-type *L. monocytogenes* was grown in low-phosphate iLSM containing H_3_^32^PO_4_ and supplemented with 1% tryptone, 2 mg/ml SHX, or both as indicated. Intracellular (p)ppGpp levels were quantified by TLC, and the mean fold ratios of (ppGpp + pppGpp)/(ppGpp + pppGpp + GTP) are reported. Data shown are representative of four independent experiments. (D) Strains of *L. monocytogenes* expressing P_*actA*__RFP were grown in iLSM in the presence or absence of 10 mM GSH and 0.5% tryptone. Fluorescence was measured and normalized to the bacterial number using the respective OD_600_. N/A denotes iLSM with no additions. Error bars show standard deviations.

The bacterial stringent response is a highly conserved, global regulatory system that responds to stress and is strongly induced during amino acid starvation (36). This response is propagated by the nucleotide secondary messenger, (p)ppGpp, which in *Firmicutes* is synthesized by the bifunctional synthase/hydrolase RelA and two accessory synthases, RelP and RelQ (37, 38). Importantly, RelA is the only identified hydrolase for (p)ppGpp in *L. monocytogenes* and is thought to be the dominant starvation-responsive synthase (39). Since CodY is canonically inactivated by high levels of (p)ppGpp during nutrient starvation, we reasoned that the addition of peptides to iLSM may prevent PrfA activation by promoting low levels of (p)ppGpp that prevent the inactivation of CodY and consequent PrfA activation. Therefore, artificially inducing the stringent response should reverse the inhibitory effect of peptides in iLSM supplemented with GSH. To induce the stringent response, DL-serine hydroxamate (SHX), which inhibits seryl-tRNA synthetase and causes accumulation of (p)ppGpp, was added to the growth media (38). During growth in iLSM, where the levels of (p)ppGpp should be naturally elevated due to limited nutrients, SHX had no discernible effect on PrfA activity under any condition (Fig. 3A). However, in iLSM medium containing tryptone, the addition of SHX with GSH was sufficient to restore full activation of the fluorescent reporter (Fig. 3B). Direct measurement of intracellular nucleotides using thin-layer chromatography (TLC) confirmed that the addition of tryptone to iLSM decreased the overall (p)ppGpp levels and that treatment with SHX was sufficient to restore the levels of (p)ppGpp (Fig. 3C).

Another strategy for artificially elevating the levels of (p)ppGpp in *L. monocytogenes* is to utilize a *relA* deletion strain that lacks the only identified (p)ppGpp hydrolase, RelA, but retains the two remaining (p)ppGpp synthases, RelP and RelQ (38). In iLSM medium with tryptone, the *relA* deletion strain was also rescued for PrfA activation by GSH, analogous to the results for the wild-type strain treated with SHX (Fig. 3D). In contrast, a mutant with a point mutation in the synthase domain of RelA (RelA^R295S^, bearing an R-to-S change at position 295) that prevents synthesis and yet allows hydrolase activity exhibited lowered levels of PrfA activation by GSH in iLSM and was no longer rescued by SHX in iLSM with tryptone (38, 40). These data suggest that high levels of (p)ppGpp are necessary and sufficient to allow activation of PrfA by GSH.

### Chemical reducing agents are sufficient to activate PrfA.

**FIG 4:**
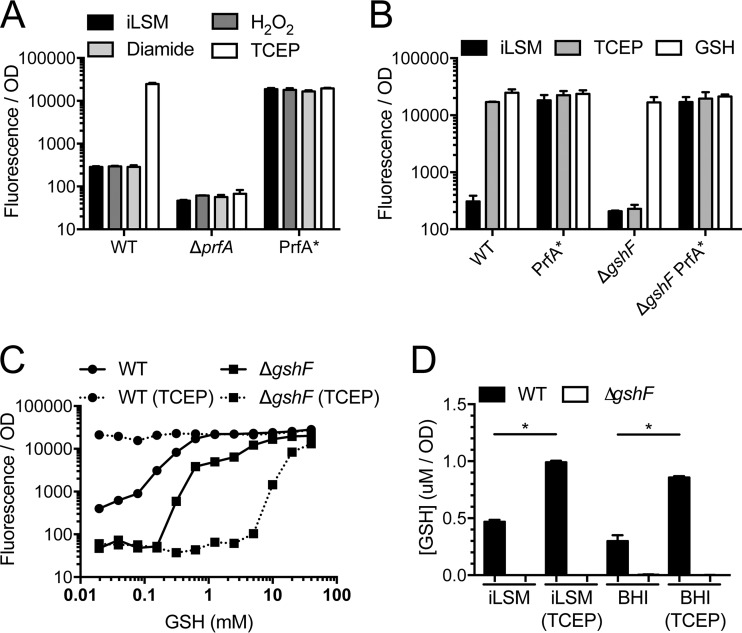
Reducing agents are sufficient to activate PrfA by increasing intracellular GSH. (A and B) Strains of *L. monocytogenes* expressing P_*actA*__RFP were grown in iLSM in the presence or absence of 100 µM hydrogen peroxide (H_2_O_2_), 0.25 mM diamide, 2 mM TCEP, or 10 mM GSH. Fluorescence was measured and normalized to the bacterial number using the respective OD_600_. (C) Wild-type or Δ*gshF L. monocytogenes* expressing P_*actA*__RFP was grown in iLSM in the presence or absence of 2 mM TCEP and various concentrations of GSH. Fluorescence was measured and normalized to the bacterial number using the respective OD_600_. (D) Wild-type or Δ*gshF L. monocytogenes* expressing P_*actA*__RFP was grown in iLSM or BHI in the presence or absence of 2 mM TCEP. Fluorescence was measured and normalized to the bacterial number using the respective OD_600_. Error bars show standard deviations. *, *P* < 0.05.

Since the addition of glutathione to iLSM was sufficient to activate PrfA *in vitro*, we considered whether other redox-related compounds would function similarly and tested iLSM supplemented with a simple oxidant (hydrogen peroxide), a thiol-specific oxidant (diamide), or a chemical reducing agent [tris(2-carboxyethyl)phosphine (TCEP)] (41, 42). Neither hydrogen peroxide nor diamide had any effect on PrfA activity; however, the addition of TCEP was sufficient to fully activate PrfA (Fig. 4A). In fact, all chemical reducing agents, including dithiothreitol and 2-mercaptoethanol, activated PrfA similarly to TCEP (J. L. Portman and D. A. Portnoy, data not shown). In support of the requirement for glutathione as a cofactor for PrfA, a mutant lacking the bacterial glutathione synthase gene *gshF* was rescued by the addition of exogenous GSH but not by TCEP (Fig. 4B) (11, 12).

Although the overall requirement of GSH for PrfA activity is clear, what was unclear was whether a small amount of exogenous GSH would be sufficient to allow a Δ*gshF* strain to respond to TCEP. To test this, GSH was titrated into cultures of wild-type and Δ*gshF* strains grown in iLSM in the presence or absence of TCEP, and PrfA activity was monitored with the reporter strain (Fig. 4C). As expected, the wild-type strain showed a dose-dependent activation of the reporter by GSH that was bypassed by the addition of TCEP. In the Δ*gshF* mutant, high levels of exogenous GSH were sufficient to activate PrfA; however, the addition of TCEP not only failed to synergize with GSH but prevented activation until higher levels of exogenous GSH were reached. These data suggested that while TCEP is sufficient to activate PrfA in a wild-type strain, it likely does so by influencing endogenously produced levels of GSH through an unknown mechanism.

Consistent with the requirement for glutathione as a cofactor for PrfA, in a mutant lacking the bacterial glutathione synthase (Δ*gshF* mutant), GSH but not TCEP activated PrfA (Fig. 4B). This result implies that the induction of PrfA by TCEP has an indirect effect on bacterial glutathione production. To test whether chemical reducing agents induce the accumulation of intracellular levels of GSH that may directly activate PrfA, the intracellular levels of GSH were measured in bacterial strains grown in iLSM after treatment with reducing agents. Exogenous TCEP indeed led to increased levels of intracellular GSH in wild-type bacteria, but not in Δ*gshF* bacteria (Fig. 4D). Elevated levels of intracellular GSH were also seen in bacteria grown in rich media despite an absence of PrfA activation under these conditions (Fig. 1A). These data support a model where exogenous GSH or reducing agents lead to increased intracellular levels of GSH; however, this increase is only sufficient for PrfA activation if the bacteria are grown in a defined medium.

### Induction of PrfA prior to infection increases virulence and immunogenicity *in vivo.*

**FIG 5:**
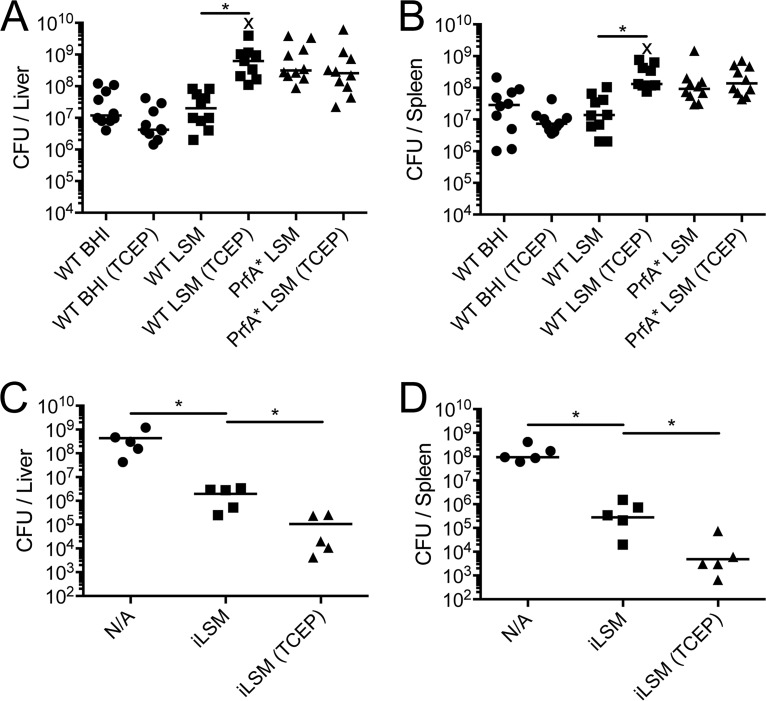
Preactivation of PrfA leads to increased bacterial burden and protection *in vivo*. (A and B) Mice were infected intravenously with 1 × 10^5^ CFU of either wild-type or PrfA* *L. monocytogenes* that was grown in iLSM or BHI with or without 2 mM TCEP prior to infection. After 48 h, the final CFU per liver and spleen were enumerated as described in Materials and Methods. *, *P* < 0.05. (C and D) Mice were injected with a PBS control or 1 × 10^3^ CFU of an attenuated strain of bacteria lacking the *actA* gene that was grown in iLSM in the presence or absence of 2 mM TCEP prior to injection. Twenty-eight to 34 days later, mice were challenged with 5 × 10^4^ CFU of wild-type bacteria, and bacterial burdens in the liver and spleen were enumerated 72 h later as described in Materials and Methods. An ‘X’ denotes the bacterial burden of a mouse that was euthanized prior to the rest of the cohort due to observable signs of medical distress, as per our animal use protocol. *, *P* < 0.05.

*L. monocytogenes* strains with constitutively active alleles of PrfA are significantly more virulent than wild-type bacteria during an acute murine infection (6). However, it is unclear at what stage(s) during infection the constitutively active allele confers an advantage (4). Utilizing iLSM supplemented with TCEP (iLSM-TCEP) to activate PrfA in wild-type *L. monocytogenes* allows early contributions of preactivated PrfA to infection to be assessed while leaving the subsequent stages of infection unperturbed. Using an intravenous (i.v.) model of infection, *L. monocytogenes* bacteria grown in iLSM-TCEP had a 60- and a 45-fold increase in the median bacterial burden in the liver and spleen, respectively, over the bacterial burdens in untreated controls at 48 h postinfection (Fig. 5A and B). There was no statistically significant difference in bacterial burdens between preactivated wild-type bacteria (iLSM-TCEP) and bacteria harboring the constitutively active allele of PrfA, PrfA*. These data suggested that the virulence advantage conferred by the PrfA* mutation occurs early during infection, likely by avoiding killing, facilitating invasion, or enabling escape from the primary vacuole.

Attenuated strains of *L. monocytogenes* are being developed as vectors for cancer immunotherapy due to their ability to elicit a robust CD8^+^ T-cell response (43–47). Increasing the immunogenicity of an attenuated vaccination strain has the potential to increase the effectiveness of these treatments or allow comparable results with a lower inoculum (48, 49). To see if preactivation of PrfA might lead to enhanced immunogenicity in a vaccination model, an attenuated strain of *L. monocytogenes* (Δ*actA* mutant) was grown in iLSM in the absence or presence of TCEP to activate PrfA prior to vaccination. Low-dose immunizations with 1,000 bacteria were performed in C57BL/6 mice intravenously, along with a phosphate-buffered saline (PBS) control. Thirty days after vaccination, mice were challenged intravenously with a lethal dose of wild-type bacteria, and after 48 h, bacterial burdens in the livers and spleens were enumerated. Pretreatment with TCEP to activate PrfA led to 10- to 100-fold decreases in bacterial burdens in the liver and spleen, signifying a significant increase in vaccination efficacy (Fig. 5C and D).

## DISCUSSION

*Listeria monocytogenes* leads a biphasic lifestyle in which it alternates between environmental saprophyte and facultative intracellular pathogen of mammals. For decades, investigators have been intrigued by how the bacteria recognize and respond to the intracellular environment of the host (4, 5, 15, 50, 51). The results of this study are consistent with a two-stage mechanism leading to the activation of PrfA, the master transcriptional regulator of *L. monocytogenes* pathogenesis. First, a metabolic licensing step is required, which can be recapitulated *in vitro* by growth in a defined synthetic medium lacking oligopeptides. Second, a reducing environment that recapitulates the redox potential of the host cytosol is required to initiate complete activation of PrfA by increasing endogenous levels of GSH, the allosteric activator of PrfA. These findings build upon and unify the literature describing virulence gene activation in *L. monocytogenes* and, furthermore, describe a simple method for complete PrfA activation *in vitro*.

Upon entering a host cell, intracellular pathogens must couple the remodeling of their metabolism with the appropriate expression of virulence factors (52). In *L. monocytogenes*, the expression and activation of PrfA is influenced by many factors, including temperature, carbon sources, l-glutamine, and BCAA levels (17, 31, 53), and yet, full activity cannot be recapitulated in complex media. The results of this study indicate that the concentrations and compositions of peptides in complex media block PrfA activation, with peptides containing BCAAs exerting the largest inhibitory effect. Our findings further suggest that the inhibition is due to the levels of (p)ppGpp, as the inhibitory effect of peptides is reversed by induction of the stringent response. Although we have not directly measured the levels of (p)ppGpp during intracellular growth, these data suggest that growth *in vivo* requires the stringent response, as observed in many pathogens (54). However, since *L. monocytogenes* can acquire amino acids from host peptides (55), it is not yet clear what stress(ors) triggers the stringent response *in vivo*. Nevertheless, *L. monocytogenes* is able to replicate rapidly *in vivo*, which implies that the cytosol contains sufficient nutrients for rapid growth. Among the many consequences of elevated (p)ppGpp is inhibition of cyclic di-AMP (c-di-AMP)-degrading phosphodiesterases, leading to elevated levels of c-di-AMP (21), which modulates central metabolism by inhibition of pyruvate carboxylase, controls osmoregulation, and triggers a host innate immune response (56, 57). Therefore, it appears that the regulation of *L. monocytogenes* metabolism, virulence regulation, and innate immunity are inextricably linked.

Another consequence of *L. monocytogenes* intracellular growth is the induction of glutathione synthase and the resulting production of glutathione, which is the allosteric activator of PrfA (11, 12). *L. monocytogenes* is one of the few Gram-positive bacteria that synthesize glutathione, where it is required for virulence and yet is dispensable in mutants in which PrfA is genetically locked into an active conformation (PrfA* mutants) (11). Thus, it appears that the primary function of glutathione during infection is PrfA activation and not its canonical role of maintaining redox homeostasis. This is not surprising, since the cytosolic environment is reducing (58). The observation that reducing agents but not oxidizing agents trigger PrfA activation suggests that *L. monocytogenes* has evolved to differentiate between the two redox stressors and utilize the uniquely reducing environment of the host cytosol as a spatiotemporal cue during pathogenesis.

There are a number of possible mechanisms to explain why the addition of reducing agents leads to an increase in intracellular GSH. In Gram-negative bacteria, the addition of reducing agents is toxic because it inhibits periplasmic disulfide bond formation, leading to extracytoplasmic stress (59). However, firmicutes like *L. monocytogenes* contain very few proteins with disulfide bonds and are therefore relatively resistant to reducing agent toxicity (60). It is more likely that the addition of reducing agents imparts reductive stress caused by altering the cellular NAD^+^/NADH balance, a condition that in Gram-positive bacteria activates a transcriptional response governed in part by the Rex transcription factor (61). We speculate that the addition of reducing agents activates Rex though an NAD^+^/NADH imbalance that either directly or indirectly leads to enhanced synthesis of GSH. Similarly, *in vivo*, the reducing nature of the host cell cytosol may lead to upregulation of *gshF* and consequent activation of PrfA.

The ability to fully activate PrfA *in vitro* allowed us to address the effect of preactivation on the overall fitness of *L. monocytogenes in vivo*. We demonstrated that stimulation of the PrfA regulon prior to acute infection led to significant increases in bacterial burdens and conferred enhanced protective immunity to subsequent lethal challenge. The increases in bacterial burdens and protective immunity are most easily explained by a boost in invasion, survival, and/or vacuolar escape. However, it is difficult to reconcile how preactivation of PrfA translates to a 45-fold increase in virulence. It is possible that the initial increase in virulence gene expression allows the bacteria to invade an alternative subset of host cells that otherwise would normally restrict replication. Further study will be necessary to delineate the exact source of the growth advantage. However, regardless of the mechanism, preactivation of PrfA clearly makes attenuated strains of *L. monocytogenes* more immunogenic and therefore has significant clinical relevance as a method to enhance the efficacy of existing therapies that utilize *L. monocytogenes* as an immunogenic platform for the treatment of cancer (47).

## MATERIALS AND METHODS

### Ethics statement.

This study was carried out in strict accordance with the recommendations in the *Guide for the Care and Use of Laboratory Animals* of the National Research Council of the National Academy of Sciences (62). All protocols were reviewed and approved by the Animal Care and Use Committee at the University of California, Berkeley (AUP-2016-05-8811).

### Bacterial strains and growth conditions.

All *L. monocytogenes* strains (Table 1) were derivatives of 10403S (63) cultured in brain heart infusion (BHI; BD Biosciences) or a defined medium specific for *L. monocytogenes* (iLSM) (56) at 37°C with shaking and without antibiotics unless otherwise stated in Materials and Methods. Growth was measured by the optical density at a wavelength of 600 nm (OD_600_). Frozen bacterial stocks were stored at −80°C in BHI plus 40% glycerol. All chemicals were purchased from Sigma-Aldrich unless otherwise stated in Materials and Methods. Antibiotics were used at the following concentrations: streptomycin at 200 µg/ml and chloramphenicol at 7.5 µg/ml for *L. monocytogenes* and 10 µg/ml for *E. coli*.

**TABLE 1:** *L. monocytogenes* strains used in this study

Strain	Description	Reference or source
10403S	Wild type	63
DP-L4317	Δ*prfA*	69
NF-L1177	PrfA* [encoded by *prfA*(G145S)]	70
DP-L6188	Δ*gshF*	11
DP-L6508	Wild type bearing pPL2_P_*actA*__RFP	23
DP-L6561	Δ*prfA* strain bearing pPL2_P_*actA*__RFP	This study
DP-L6562	PrfA* strain bearing pPL2_P_*actA*__RFP	This study
DP-L6563	Δ*gshF* strain bearing pPL2_P_*actA*__RFP	This study
DP-L6291	RelA^R295S^	38
DP-L6292	Δ*relA*	38
DP-L3078	Δ*actA*	71

### Growth supplements and inhibitors.

For supplementation with purified peptides, 0.075 g hexapeptide (0.025% [wt/vol] final concentration; 49.7 mM for KGGGGK, 44.7 mM for KAAAAK, and 34.4 mM for KIIIIK and KLLLLK) was dissolved in 3 ml iLSM and the LSM-peptide product was sterilized using a 0.2-µm filter. Specific peptides were purchased from Mimotopes (Australia) and had specifications as follows: KGGGGK (MW = 502.57 g mol^−1^, >65% pure), KAAAAK (MW = 558.68 g mol^−1^, >61% pure), KIIIIK (MW = 727.00 g mol^−1^, >71% pure), and KLLLLK (MW = 727.00 g mol^−1^, >72% pure). dl-Serine hydroxamate was used where noted at a final concentration of 2 mg/ml.

### Fluorescence reporter assay.

Strains of *L. monocytogenes* harboring the integrating plasmid pPL2 expressing RFP controlled by the *actA1p* promoter (referred to herein as P_*actA*__RFP) were grown overnight at 37°C in iLSM with shaking (23). These cultures were diluted 1:10 into the noted media with any applicable supplements and grown at 37°C with shaking until reaching an OD_600_ of approximately 2. Five hundred microliters was taken from each culture, transferred into a clear 24-well flat-bottom plate, and subsequently read for fluorescence intensity on a Tecan M1000 multiplate reader with the following parameters: 560/580 (excitation/emission), bottom read, optimal flashes, optimal gain. The OD_600_ was taken with a handheld spectrophotometer in parallel for normalization.

### RT-qPCR of bacterial transcripts.

Bacteria were grown overnight and subcultured 1:20 into 5 ml iLSM. Bacteria were harvested at an OD_600_ of 1.0 by the addition of an equal volume of RNAprotect bacterial reagent (Qiagen). Bacteria were harvested by centrifugation and flash-frozen in liquid nitrogen prior to RNA extraction. Bacteria were lysed in phenol-chloroform containing 1% SDS by vortexing with 0.1-mm-diameter silica-zirconium beads (BioSpec Products, Inc.). Nucleic acids were precipitated from the aqueous fraction overnight at −80°C in ethanol containing 150 mM sodium acetate (pH 5.2). Precipitated nucleic acids were washed with ethanol and treated with Turbo DNase according to the manufacturer’s instructions (Life Technologies Corporation). RNA was again precipitated overnight and then washed in ethanol. RT-PCR was performed with iScript reverse transcriptase (Bio-Rad), and quantitative PCR (qPCR) of the resulting cDNA was performed with Kapa SYBR fast (Kapa Biosystems) using the manufacturer’s recommended cycling parameters. The primers used for qPCR of *actA* transcripts were as follows: *actA*_F, CGACATAATATTTGCAGCGAC, and *actA*_R, TGCTTTCAACATTGCTATTAGG.

### Immunoblotting for LLO.

Briefly, overnight cultures of bacteria in iLSM were diluted 1:10 into iLSM in the presence or absence of 10 mM GSH and incubated for 6 h at 37°C with shaking, and then the bacteria were separated from the supernatant by centrifugation. The supernatant was treated with 10% (vol/vol) trichloroacetic acid (TCA) for 1 h on ice to precipitate protein. The protein pellet was washed twice with ice-cold acetone, followed by vacuum drying. The proteins were dissolved in lithium dodecyl sulfate (LDS) buffer (Invitrogen) containing 5% β-mercaptoethanol (BME), using a volume that normalized for the OD_600_ of harvested bacteria, and then were boiled for 10 min and separated by SDS-PAGE. The primary antibodies, a rabbit polyclonal antibody against LLO and a mouse monoclonal antibody against P60 (Adipogen), were each used at a dilution of 1:5,000. P60 is a constitutively expressed bacterial protein used as a loading control for secreted proteins (64). All immunoblots were visualized and quantified using the Odyssey imager and appropriate secondary antibodies from the manufacturer according to the manufacturer’s instructions.

### (p)ppGpp quantification.

(p)ppGpp was measured as previously described with minor changes (38, 65). Bacteria were grown in low-phosphate listeria synthetic medium (LPLSM) (56), to which phosphate was added at a dilution of 1/2,000 (1/20 of the normal concentration). Bacterial overnight cultures were diluted into LPLSM and grown for 2 to 5 h before a secondary dilution of 5 × 10^8^ bacteria was made into 100 µl of either LPLSM or LPLSM plus 1% Bacto–tryptone with 20 µCi/ml carrier-free H_3_^32^PO_4_. These cultures were incubated for 120 min at 37°C before resuspending in 50 µl of 13 M formic acid and freeze-thawing 4 times in a dry ice-ethanol bath to lyse the cells. When utilized, serine hydroxamate was added at a concentration of 2 mg/ml for the final 15 min before harvest. Cell debris was removed by centrifugation, and extracts were spotted onto polyethyleneimine (PEI) cellulose thin-layer chromatography (TLC) plates (EMD Millipore) and developed in 1.5 M KH_2_PO_4_, pH 3.4. Dried TLC plates were exposed to phosphor-storage screens (Kodak) for >4 h before imaging on a Typhoon scanner (GE Healthcare). Nucleotides were identified using [γ-^32^P]GTP and *E. coli* wild-type standard CF1943 (W3110 parental strain), which was generously provided by Michael Cashel (National Institutes of Health). The phosphor-storage screen scan results were quantified using ImageJ software (National Institutes of Health) without background subtraction. The volumes of intensity (without background correction) for identified nucleotide spots were used for calculation of (p)ppGpp levels as follows: (pppGpp + ppGpp)/(pppGpp + ppGpp + GTP).

### Intracellular glutathione quantification.

Reduced glutathione (GSH) and oxidized glutathione (GSSG) concentrations were measured by using a commercial kit supplied by Cayman Chemical according to the manufacturer’s recommendations and as described previously (66). Briefly, bacteria were grown to mid-log phase in either iLSM or BHI and resuspended in PBS containing 1 mM EDTA at a pH of 6.5. Bacteria were lysed by vortexing with 0.1-mm-diameter silica-zirconium beads (BioSpec Products, Inc.), and the lysate was stored on ice. Samples were deproteinated with an equal volume of meta-phosphoric acid and stored at −20°C prior to quantification with the supplied kit.

### *In vivo* infections.

For acute infections, 8- to 12-week-old female C57BL/6 mice (The Jackson Laboratory) were infected intravenously with 1 × 10^5^ CFU in 200 µl of PBS as described previously (67, 68), and organs were harvested 48 h later. For immunization studies, mice were injected with 1 × 10^3^ CFU in 200 µl of PBS of an attenuated strain of *L. monocytogenes* harboring a deletion in the *actA* gene. Twenty-eight to 34 days later, mice were challenged with 5 × 10^4^ CFU of wild-type *L. monocytogenes* in 200 µl of PBS, and organs were harvested 72 h later. In both cases, all bacteria were grown to an OD_600_ of approximately 0.5 in either iLSM or BHI in the presence or absence of 2 mM TCEP. The bacteria were washed twice with PBS, suspended in a solution of 9% glycerol in PBS, and then flash frozen in liquid nitrogen before storage at −80°C. Prior to infection, frozen bacteria were thawed and diluted to the appropriate cell density in PBS and plated for enumeration in parallel with the infection to verify inoculum accuracy. To collect organs, the mice were euthanized and spleens and livers were harvested, homogenized in 5 ml or 10 ml IGEPAL CA-630 (Sigma), respectively, and plated for enumeration of bacterial burdens.

### Statistical analysis.

Statistical analyses were carried out with the GraphPad Prism software (version 7.0a). Values plotted exponentially were transformed to base 10 logarithmic values before being used for statistical analyses. One-way ANOVA with Tukey’s *post hoc* test was used to compare groups, with only relevant comparisons noted on each figure for clarity.
